# Cadmium induces Wnt signaling to upregulate proliferation and survival genes in sub-confluent kidney proximal tubule cells

**DOI:** 10.1186/1476-4598-9-102

**Published:** 2010-05-08

**Authors:** Prabir K Chakraborty, Wing-Kee Lee, Malte Molitor, Natascha A Wolff, Frank Thévenod

**Affiliations:** 1 ZBAF, Department of Physiology & Pathophysiology, University of Witten/Herdecke, Stockumer Strasse 12, D-58453 Witten, Germany

## Abstract

**Background:**

The class 1 carcinogen cadmium (Cd^2+^) disrupts the E-cadherin/β-catenin complex of epithelial adherens junctions (AJs) and causes renal cancer. Deregulation of E-cadherin adhesion and changes in Wnt/β-catenin signaling are known to contribute to carcinogenesis.

**Results:**

We investigated Wnt signaling after Cd^2+^-induced E-cadherin disruption in sub-confluent cultured kidney proximal tubule cells (PTC). Cd^2+ ^(25 μM, 3-9 h) caused nuclear translocation of β-catenin and triggered a Wnt response measured by TOPflash reporter assays. Cd^2+ ^reduced the interaction of β-catenin with AJ components (E-cadherin, α-catenin) and increased binding to the transcription factor TCF4 of the Wnt pathway, which was upregulated and translocated to the nucleus. While Wnt target genes (*c-Myc*, *cyclin D1 *and *ABCB1*) were up-regulated by Cd^2+^, electromobility shift assays showed increased TCF4 binding to *cyclin D1 *and *ABCB1 *promoter sequences with Cd^2+^. Overexpression of wild-type and mutant TCF4 confirmed Cd^2+^-induced Wnt signaling. Wnt signaling elicited by Cd^2+ ^was not observed in confluent non-proliferating cells, which showed increased E-cadherin expression. Overexpression of E-cadherin reduced Wnt signaling, PTC proliferation and Cd^2+ ^toxicity. Cd^2+ ^also induced reactive oxygen species dependent expression of the pro-apoptotic ER stress marker and Wnt suppressor CHOP/GADD153 which, however, did not abolish Wnt response and cell viability.

**Conclusions:**

Cd^2+ ^induces Wnt signaling in PTC. Hence, Cd^2+ ^may facilitate carcinogenesis of PTC by promoting Wnt pathway-mediated proliferation and survival of pre-neoplastic cells.

## Background

Wnts are secreted to activate signaling processes controlling cell proliferation and body patterning throughout development. Though there are several branches of the Wnt-mediated signaling cascade in mammals the most prominent is the canonical Wnt pathway [1,2]. Its hallmark is the accumulation of the junctional protein β-catenin in the cytoplasm, which then translocates to the nucleus to trigger the β-catenin/T-cell factor/lymphoid enhancer factor (TCF/LEF) transcriptional machinery, and upregulate target genes, such as *cyclin D1*, *c-Myc *and *ABCB1*. Under normal conditions, β-catenin is marked for degradation by a multi protein degradation ("destruction") complex, which maintains its levels low in the cytoplasm through continuous degradation by the 26S ubiquitin-proteasome pathway. The tumor suppressor protein Axin acts as the scaffold of this complex by directly interacting with adenomatous polyposis coli (APC), glycogen synthase kinase 3-β (GSK3-β), casein kinase 1-α (CK1-α) and β-catenin. Conversely, this process is regulated by the Wnt signaling cascade which inhibits GSK3-β and thus β-catenin degradation [1,2].

Interestingly, in the cell β-catenin has two functions: (i) as a latent signaling molecule as part of the Wnt signaling pathway; and (ii) as a structural protein in adherens-junctions (AJs), participating in cell-cell adhesion by bridging E-cadherin to α-catenin. The cadherins are Ca^2+^-dependent cell adhesion glycoproteins that physically link neighboring cells together [3]). The development of AJs then enables the establishment of functional tight junctions (TJs) which are responsible for the regulation of the paracellular epithelial permeability [4]. The disruption of cadherin-catenin complexes causes an increase in nuclear β-catenin/TCF-mediated transcription of Wnt responsive genes [5]. In contrast, stabilization of the cadherin-catenin complex shows reduced β-catenin mediated Wnt signaling [6].

Deregulation of E-cadherin adhesion is a crucial step during tumor cell migration, invasion and metastasis; therefore many epithelial cancer cells repress E-cadherin expression [7]. By contrast, disruption of E-cadherin cell-cell adhesion in normal tissues induces growth arrest and cell death [8]. Changes of the Wnt/β-catenin signal cascade can also contribute to the development of cancers. Furthermore, mutations in the Wnt pathway lead to 90% of colon cancers and also to cancers of the lungs, kidney, liver, etc. (reviewed in [9]). The expression of the TCF isoform, TCF4 is highest in organ sites with active Wnt signaling like the central nervous system and intestinal epithelium, but also kidney. Hence loss of cell-cell adhesion and uncontrolled Wnt signaling promote cancer induction and progression.

Cadmium and cadmium compounds are group 1 human carcinogens [10]. The evidence for carcinogenicity in humans is also supported by recent epidemiologic evidence indicating that cadmium induces cancer in many organs in humans, including the kidneys [11,12]. The present consensus is that a direct mutagenic effect of cadmium is weak [13], but is presumably sufficient to induce tumors if combined with other pro-carcinogenic effects of cadmium, such as formation of reactive oxygen species (ROS) and/or interference with anti-oxidative enzymes, inhibition of DNA repair enzymes, deregulation of cell proliferation, interference with the balance between pro and anti-apoptotic mechanisms, and disruption of cell adhesion [14].

One early event associated with cadmium ion (Cd^2+^) nephrotoxicity is the alteration of the properties of AJs and TJs, most likely due to Ca^2+ ^displacement, which causes disruption of the homophilic E-cadherin interaction [15]. This causes a loss of integrity in the cell-cell adhesion belt and disassembly of TJs with a concomitant decrease in the trans-epithelial resistance and increased paracellular permeability (reviewed in [16]). In a recent study, we confirmed that micromolar Cd^2+ ^concentrations decrease trans-epithelial resistance of cultured WKPT-0293 Cl.2 rat kidney proximal tubule cells (PTC) within 1 hour of exposure which correlated with a decrease of membrane-associated E-cadherin and β-catenin and an increase of *c-Myc *and *Abcb1 *mRNA after 3 hours [17]. Hence, the present study sought to investigate the impact of disruption of AJs by Cd^2+ ^on β-catenin/Wnt signaling in cultured kidney PTC. The data demonstrate that Cd^2+ ^induces Wnt signaling in PTC to upregulate proliferation and survival genes.

## Methods

### Materials and antibodies

Cycloheximide and actinomycin D were purchased from Roth (Karlsruhe, Germany), lactacystin, rabbit serum (cat. #S2632) and protease inhibitor cocktail were obtained from Sigma-Aldrich (Deisenhofen, Germany). Recombinant protein G sepharose 4B was from Invitrogen (Karlsruhe, Germany). Antibodies (all mouse monoclonal) used were: anti-β-catenin (cat. # 610153; 1:1000) and anti-E-cadherin (cat. #610181; 1:2500) from BD Biosciences (Heidelberg, Germany), anti-α-catenin (cat. # ab19446; 1:1000) from Abcam (Cambridge, UK), anti-CHOP (clone9C8) (cat. # MA1-250; 1:1000) from Dianova GmbH (Hamburg, Germany), anti-TCF4 (clone6H5-3) (cat. # 05-511; 1:1000) from Millipore GmbH (Schwalbach, Germany), anti-GAPDH (cat. # G8795; 1:20,000), anti-β-actin (cat. # A5316; 1:10,000) and anti-γ-tubulin (cat. # T6557; 1:10,000) from Sigma-Aldrich. The secondary sheep anti-mouse horseradish peroxidase (HRP)-linked IgG (NA931; 1:5000) was purchased from Amersham Biosciences (Freiburg, Germany). All other chemicals were of the highest purity grade available.

### Cell culture

Cells from the S1-segment of rat PT (WKPT-0293 Cl.2) were immortalized by retroviral transfection with SV40 large T-antigen [18] and cultured as previously described [19] at 37°C in 5% CO_2_. Apart from some ECIS experiments (see below), all Cd^2+ ^experiments were performed in serum-free medium (SFM). Unless otherwise indicated, 25 μM Cd^2+ ^was used. For immunoblot experiments comparing subconfluent and confluent cells ~50% confluence was obtained by seeding 5 × 10^5 ^cells per well and 100% confluence by seeding 1 × 10^6 ^cells per well in 6-well plates. After 48 hours WKPT-0293 Cl.2 cells were incubated in the absence or presence of Cd^2+ ^in SFM before harvesting.

### Preparation of cytosolic and nuclear protein extracts

The protocol was adapted from the method described by Andrews and Faller [20]. Cells were harvested into 400 μl of ice cold buffer A (10 mM HEPES-KOH, pH 7.9, 1.5 mM MgCl_2_, 10 mM KCl, 0.5 mM DTT, 0.05% Nonidet P-40, 0.2 mM Pefabloc SC and a protease inhibitor cocktail), and allowed to lyse on ice for 10 min. After a pre-run at 4°C at 500 × g for 1 min to remove unlysed cells and debris, further centrifugation was performed at 16,000 × g for 1 min. The supernatant containing cytosolic proteins was collected. Exactly 100 μl of buffer B (20 mM HEPES-KOH, pH 7.9, 25% glycerol, 420 mM NaCl, 1.5 mM MgCl_2_, 0.2 mM EDTA, 0.5 mM DTT, 0.05% Nonidet P-40, 0.2 mM Pefabloc SC and protease inhibitor cocktail), was added to the pellet and incubated on ice for 20 min. The suspension was centrifuged at 16,000 × g for 2 min and the supernatant containing the nuclear proteins was collected.

### Immunoblotting

Protein concentration of samples was determined by the Bradford method [21], using bovine serum albumin as a standard. SDS-PAGE and transfer were performed exactly as described elsewhere [17]. Signals were quantified using ImageJ or TINA 2.09 software. For comparisons between control and experimental conditions specific signals were first normalized to loading markers (β-actin, γ-tubulin or GAPDH).

### Immunoprecipitation

All steps were performed at 4°C. Cells (150-300 μg) were lysed in 250 μl of RIPA buffer (50 mM Tris-HCl, pH 8.0, 150 mM NaCl, 1% Nonidet P-40, 0.5% sodium deoxycholate, 0.1% SDS) and 10 μl protease inhibitor cocktail). The solution was centrifuged at 15,800 × *g *to remove particulate matter. Lysate containing 150 μg of protein was pre-cleared with rabbit serum and recombinant protein G sepharose 4B, mixed with 4 μg/ml of antibody to β-catenin or TCF4 for 16 hours at 4°C and then collected using protein G sepharose 4B by incubation for 30 min. Immunoprecipitates were washed three times with RIPA buffer. After centrifugation, the pellets were resuspended in sample buffer and heated for 5 min at 95°C for SDS-PAGE, followed by immunoblot analysis.

### Laser scanning confocal and immunofluorescence microscopy

Cells (5 × 10^4^) were grown in 24-well plates on glass cover-slips. After treatment coverslips were washed with PBS, fixed with 4% paraformaldehyde in PBS, permeabilized with 0.2% TritonX-100 for 5 min, and blocked with 1% BSA and 100 μg/ml RNAse A for 2 h. Thereafter, the cells were incubated with primary antibodies against β-catenin (1:1000), TCF4 (1:250) or E-cadherin (1:1000) followed by staining with anti-mouse Alexa-Fluor 488 conjugated secondary antibody (1:400) (Invitrogen GmbH; Karlsruhe, Germany) for 1 h and subsequent propidium iodide (PI) staining (10 μg/ml) for 5 min. Then the cells were washed and samples were visualized with a LEICA TCS SP5 confocal laser microscope (Wetzlar, Germany) by exciting at 488 nm (emission 520 nm) to detect β-catenin and TCF4, and at 543 nm (emission 617 nm) for PI. E-cadherin overexpressing and mock-transfected cells were viewed with a Zeiss Axiovert 200 M microscope (Carl Zeiss, Jena, Germany) using filters for FITC with excitation/emission wavelengths of 480/535 nm, respectively. Images were acquired at fixed exposure times (600 ms), processed, and analyzed semiquantitatively with MetaMorph software (Universal Imaging Corporation, Downingtown, PA).

### Electrophorectic mobility shift assay (EMSA)

Nuclear fractions were used for EMSA of TCF4 binding to *cyclin D1 *and *ABCB1 *promoters. Based on the consensus TCF/LEF-binding motifs, CTTTGA/TA/T [22] the wild-type TCF/LEF binding sequence of the human *cyclin D1 *promoter 5'-CTCTGCCGGGCTTTGATCTTTGCTTAACA-3' [23] and the binding sequence of human *ABCB1 *5'-GG**CTTTGAA**GTATGA-3' [24] were selected. They were end-labeled with [γ-^32^P] dATP by incubating oligodeoxyribonucleotide strands with 5× reaction buffer and 10 U T4 polynucleotide kinase (Fermentas, St. Leon-Rot, Germany) for 1 h at 37°C. Then labeled oligonucleotides were allowed to anneal at room temperature for 10 min and 20 μg protein from each sample was used in 25 μl binding reactions, which consisted of 1 μg poly dI-dC, in 5× binding buffer (50 mM Tris HCl;pH 8.0, 750 mM KCl, 2.5 mM EDTA, 0.5% Triton-X 100, 62.5% glycerol (v/v) and 1 mM DTT). To determine specificity of DNA binding, samples were incubated with or without 20 ng of unlabeled competitor DNA for 10 min at room temperature. Then 0.1 ng of labeled probe was added and samples were further incubated for 20 min at room temperature. Samples were separated on a 5% non-denaturing polyacrylamide gel in 0.5% TBE and visualized by autoradiography.

### RT-PCR

Total RNA was extracted using the RNeasy Mini Kit (Qiagen, Hilden, Germany) and first strand cDNA was synthesized with the Omniscript RT kit (Qiagen), using 1 μg of RNA per 20 μl reaction and oligo(dT) primer. cDNA was then utilized in PCR reactions for *c-Myc *and *GAPDH *as previously described [17]. Remaining primers are summarized in the Additional file 1.

Following activation at 95°C for 15 min, the following PCR conditions for each primer pair were performed: For *Lef-1*, *TCF-3*, *TCF-4*, *E-cadherin *and *β-catenin *28 cycles at 94°C for 20 s, 62°C for 30 s, 72°C for 45 s; for *cyclin D1 *34 cycles at 94°C for 30 s, 56°C for 60 s, 72°C for 90 s; for *Abcb1a *32 cycles at 95°C for 80 s, 56°C for 60 s and 72°C 90 s; for *CHOP *28 cycles at 94°C for 30 s, 62°C for 30 s and 72°C for 45 s.

### Plasmids and transient transfections

Myc-epitope-tagged full length human TCF4 and a deletion mutant lacking the NH_2_-terminal 30 amino acids (ΔN-hTCF4), which are essential for its interaction with β-catenin, were inserted in the expression vector pcDNA3 [25] and were a gift of Dr. Bert Vogelstein (Baltimore, Maryland, USA). Full-length E-cadherin inserted into the plasmid pL31NU [26] was a gift of Dr. Rolf Kemler (Freiburg Germany), the full length mouse CHOP construct in pcDNA1 was a gift of Prof. David Ron (New York, NY, USA) and the ΔN-β-catenin construct in pCGN lacking the NH_2_-terminal stretch of 132 amino acids required for its degradation [27] was a gift of Prof. Avri Ben-Ze'ev (Rehovot, Israel).

Unless otherwise indicated, WKPT-0293 Cl.2 cells (5 × 10^5 ^cells per well in 6-well plates) were transiently transfected 24 h post-seeding with the various constructs using Lipofectamine 2000 reagent (Invitrogen) following manufacturer's instructions. Plasmids and Lipofectamine reagent were used at a constant ratio of 1:2.5 and incubated for up to 36 h.

### Luciferase assay

Wnt signaling was assessed using the well-described TOPflash assay [25]. Briefly, 5 × 10^4 ^cells per well grown for 24 h in 24-well plates were transiently transfected with TOPflash or FOPflash (Millipore) using poly(ethyleneimine) (PEI; Sigma) from a stock solution of 1 mg/ml at a ratio of 1:0.75 (volume DNA:volume PEI). For experiments with ΔN-β-catenin, cells were first transfected with ΔN-β-catenin construct and then with TOPflash or FOPflash 6 h later. Luciferase activity was determined using Tropix Luminescence assay kit (Applied Biosystems Applera Deutschland GmbH, Darmstadt, Germany) after 24 h and the measurements were performed in a Mithras LB 940 multimode microplate reader (Berthold Technologies GmbH, Bad Wildbad, Germany). The readings were normalized against protein concentration determined by the Bradford method [21] for each sample. For experiments with ~50% confluent or 100% confluent cells 5 × 10^4 ^and 9 × 10^4 ^cells per well, respectively, were grown in 24-well plates. The degree of cell confluence refers to the time of transfection with TOPflash or FOPflash.

### MTT toxicity assay

The 3-(4,5-dimethylthiazol-2-yl)-2,5-diphenyltetrazolium bromide (MTT) method was modified as previously described [17] and measured on a Helios Epsilon spectrophotometer (Thermo Scientific, Langenselbold, Germany). The values were normalized to the control, which was equivalent to 100% cell viability, to determine cell death rates. To account for the loss of cells during the transfection procedure, different cell numbers (1.5 × 10^4 ^for empty vector and CHOP, 2.5 × 10^4 ^for E-cadherin in 48 well plates) were plated to obtain a similar cell density prior to treatment with Cd^2+^.

### Electric cell-substrate impedance sensing (ECIS)

Eighteen to 24 hours after transfection, WKPT-0293 Cl.2 cells were trypsinized, centrifuged at 400 × g for 5 min, and plated in ECIS 8W10E cell culture arrays at 3.5 - 5.5 × 10^5 ^cells per well. For experiments evaluating the effect of Cd^2+ ^on the attachment and proliferation of CHOP-transfected cells serum-containing medium (SCM) included 50 μM CdCl_2_. The changes in resistance (R) and capacitance (C) of the recording electrodes were determined using an ECIS™1600R instrument (Applied BioPhysics, Troy, NY), as previously described [17]. C_40 kHz _and R_400 Hz _values reflect electrical properties of epithelial monolayers due to cell attachment/spreading/proliferation and barrier formation, respectively. Whereas C_40 kHz _mirrors attachment and spreading of cells R_400 Hz _values are indicative of the epithelial barrier integrity, especially establishment of tight intercellular contacts. For experiments evaluating cell attachment and proliferation, the cells were incubated in SCM, with twice daily medium renewal. Once the cells had established a functional monolayer with stable R and C readings, they were washed once with SFM and the medium was replaced with SFM ± 20 μM Cd^2+^.

### Determination of cellular levels of reactive oxygen species (ROS)

The nonfluorescent, dye 5-(and-6)-carboxy-2',7'-dichlorodihydrofluorescein diacetate (carboxy-H_2_DCFDA) is oxidized to fluorescent carboxydichlorofluorescein by H_2_O_2_, peroxyl radicals and peroxynitrite anion (Invitrogen GmbH; Karlsruhe, Germany). WKPT-0293 Cl.2 cells (2 × 10^5^; 6-well plates) were loaded with 20 μM carboxy-H_2_DCFDA, in the presence of the ABCB1 inhibitor PSC833 (1 μM) to improve dye loading, for 30 min at 37°C. After washing, cells were gently trypsinized, pelleted by centrifugation at 400 × g for 3 min, brought into suspension, and added to each well of a 96 well plate ± Cd^2+^. Data were collected every 60 sec at λ_ex_/λ_em _of 500/535 nm on a Berthold Mithras LB940 fluorescence microplate reader. When used, cells were pre-incubated with 100 μM α-tocopherol for 1 h prior to dye loading and remained in the presence of α-tocopherol for the duration of the measurements.

### Statistics

Unless otherwise indicated, the experiments were always repeated at least three times with independent cultures. Means ± SEM are shown, unless otherwise indicated. Statistical analysis using unpaired Student's t-test was carried out with Sigma Plot 8.0 (Inc., Chicago, IL). For more than two groups, one-way ANOVA was used assuming equality of variance with Levene's test and Tukey post hoc test for pair-wise comparison with SPSS 12.0. Results with *P *< 0.05 were considered to be statistically significant.

## Results

### Cd^2+ ^disrupts the E-cadherin-catenin adherens junction complex and causes translocation of β-catenin to cytosol and nuclei of kidney PTC

**Figure 1:**
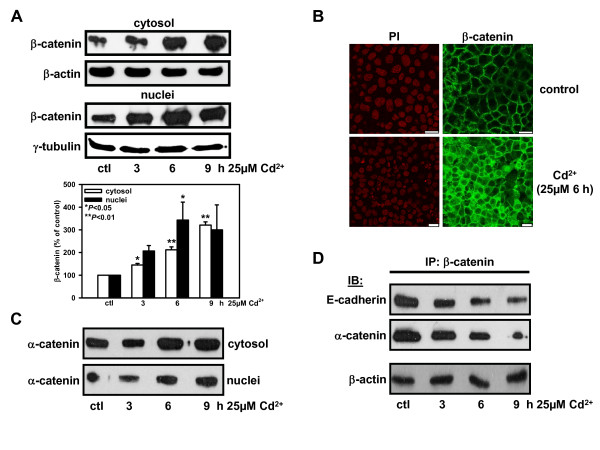
**Cd^2+ ^disrupts the adherens junction complex and redistributes β-catenin from the periphery to cytosol and nuclei of kidney PTC**. (A) Time dependent increase of β-catenin protein distribution in cytoplasmic and nuclear fractions of WKPT-0293 Cl.2 cells without (ctl) or with Cd^2+^. Cytosolic and nuclear fractions were immunoblotted with β-catenin antiserum. For loading controls, the same membranes were reprobed with antibodies to β-actin and γ-tubulin for cytoplasmic and nuclear fractions, respectively. β-catenin signals were normalized to loading markers and compared to controls, which were set to 100%. Means ± SEM of 3 experiments are shown. One-way ANOVA was used assuming equality of variance with Levene's test and Tukey post hoc test for pair-wise comparison between control and Cd^2+ ^exposed cells. (B) Immunofluorescence staining patterns of β-catenin in WKPT-0293 Cl.2 cells ± Cd^2+^. Nuclei were stained with propidium iodide (PI). Note the peripheral β-catenin labeling in control cells whereas in Cd^2+ ^exposed cells β-catenin is found diffusely distributed in cytosol and nuclei. Bars = 20 μm. (C) Distribution of α-catenin in cytoplasmic and nuclear fractions increases with Cd^2+ ^exposure time, as shown by immunoblotting. (D) Cd^2+ ^reduces association of β-catenin with E-cadherin and α-catenin as a function of time. Cell lysates were immunoprecipitated (IP) with β-catenin antiserum and immunoblotted (IB) with E-cadherin or α-catenin antisera.

One early event associated with Cd^2+ ^nephrotoxicity is disruption of the homophilic E-cadherin interaction [15,16]. In the present study, exposure of sub-confluent WKPT-0293 Cl.2 cells to Cd^2+ ^(25 μM in SFM) for 3-9 h led to cytoplasmic and nuclear accumulation of β-catenin (Fig. 1A), indicating disruption of the AJ complex. Statistical analysis of 3 different experiments displays significant β-catenin increase in cytosol already after 3 h and in nuclei after 6 h. This was corroborated by confocal immunofluorescence microscopy of Cd^2+ ^treated PTC which exhibited a more diffuse β-catenin distribution in the cytoplasm and nucleus compared to the controls where β- catenin was predominately found at the cell borders (Fig. 1B).

A possible explanation for the higher levels of β-catenin is increased synthesis induced by Cd^2+^. However, mRNA levels of β-catenin were unchanged by Cd^2+ ^(Additional file 2a) and the translation inhibitor cycloheximide had no effect on Cd^2+^-induced β-catenin redistribution (Additional file 2b). To find out whether the increase in β-catenin might be due to inhibition of the proteasomal degradation complex by Cd^2+^, we utilized lactacystin, a potent proteasomal inhibitor. After 6 h the increase of the cytosolic and nuclear pool of β-catenin observed with Cd^2+ ^was not mimicked by lactacystin (Additional file 2c), indicating that Cd^2+ ^does not inhibit the proteasome. Thus the elevation of β-catenin must be a consequence of redistribution of β-catenin from the membrane pool to cytosol and nuclei.

Alpha-catenin is another member of the AJ complex assembly, which mediates interaction between AJs and actin filaments, therefore disruption of the complex should also lead to the release of α-catenin. Similarly to β-catenin, α-catenin content in the cytoplasm and nuclei steadily increased under Cd^2+ ^treatment of sub-confluent WKPT-0293 Cl.2 cells at 3-9 h (Fig. 1C). β-catenin/TCF/LEF1 mediated transcription has been reported to be inactivated through β-catenin binding to E-cadherin [28] or α-catenin [29]. However, binding of β-catenin to E-cadherin as well as α-catenin is reduced by Cd^2+ ^exposure (Fig. 1D), supporting the disruptive action of Cd^2+ ^on the AJ and negating the probability of transcriptional inactivation of β-catenin by α-catenin.

### Cd^2+ ^exposure induces translocation of TCF4 to the nucleus and activation of the canonical Wnt/β-catenin pathway in PTC

**Figure 2:**
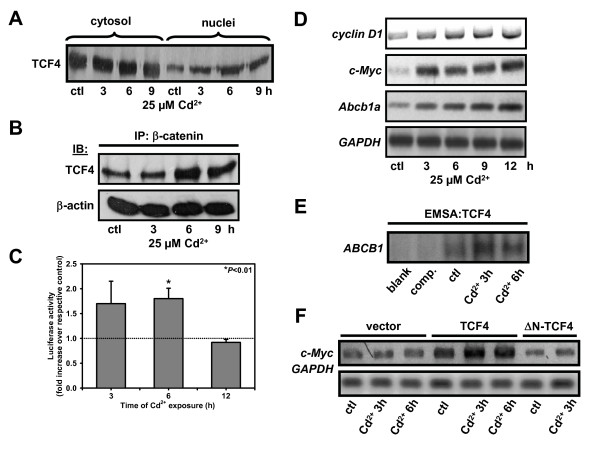
**Cd^2+ ^triggers nuclear translocation of TCF4 and activates TCF4/β-catenin mediated transcription of cell proliferation and survival genes in kidney PTC**. (A) Distribution of TCF4 protein in cytoplasmic and nuclear fractions of WKPT-0293 Cl.2 cells ± Cd^2+ ^was determined by immunoblotting. (B) Increased association of immunoprecipitated β-catenin with TCF4 by Cd^2+ ^in WKPT-0293 Cl.2 cells. (C) Cd^2+ ^induces TCF transcriptional activity. Luciferase activity of TOPflash or FOPflash transfected cells treated with 25 μM Cd^2+ ^for 3-12 h. Data were corrected for protein and normalized to controls at each time point. Means ± SEM (n = 3-8) are shown. Student's unpaired *t*-test compares Cd^2+ ^treated cells to respective controls. (D) Effect of Cd^2+ ^exposure on mRNA of Wnt pathway target genes. *GAPDH *was used as housekeeping gene. (E) EMSA of TCF4 binding to the *ABCB1 *promoter region. Extracts from controls (ctl), Cd^2+ ^(25 μM) treated cells, cells exposed to 3 h Cd^2+ ^followed by incubation with a 200-fold excess of competing unlabeled oligonucleotides (comp.), and lysate-free sample (blank) were loaded. Binding of oligonucleotides to TCF4 was enhanced upon Cd^2+ ^exposure. (F) Effect of TCF4 overexpression on Cd^2+ ^induced *c-Myc *mRNA expression. WKPT-0293 Cl.2 cells transiently transfected with full length human TCF4 (TCF4), human TCF4 lacking the interaction domain for β-catenin (ΔN-TCF4), or empty vector were treated with ± 25 μM Cd^2+^. TCF4 overexpression enhanced basal and Cd^2+^-induced *c-Myc *expression, whereas ΔN-TCF4 had no effect.

The pivotal transcription factor for the canonical Wnt pathway, the TCF4 protein, is responsible for transactivation of cell proliferation and survival genes. Strikingly, we observed increased localization of TCF4 in nuclei upon Cd^2+ ^exposure from an early time point of 3 h and remained elevated up to 9 h (Fig. 2A). This could also be visualized in confocal laser scanning micrographs (data not shown). A prerequisite for activation of the Wnt pathway-mediated expression of target genes is binding of β-catenin to TCF4 in the nucleus. To evidence this, immunoprecipitation was performed with β-catenin and bound TCF4 was determined by immunoblotting. Cd^2+^-treated samples exhibited more β-catenin-TCF4 binding as early as 3-6 h (Fig. 2B). To confirm β-catenin/TCF4 interaction, we repeated the experiment at 3-9 h Cd^2+ ^incubation by pulling down TCF4 and immunoblotting for β-catenin (Additional file 3a) thus establishing that there is significantly more β-catenin bound to TCF4 under Cd^2+ ^treatment conditions.

**Figure 3:**
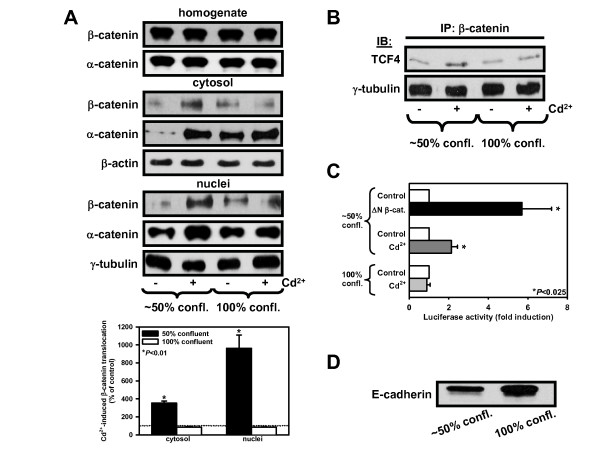
**Cd^2+^-induced nuclear translocation of β-catenin and Wnt signaling are abolished in confluent kidney PTC, which show increased E-cadherin expression**. (A) Immunoblots with β-catenin or α-catenin antisera in ~50% and 100% confluent WKPT-0293 Cl.2 cells (25 μM Cd^2+^, 6 h). For statistical analysis of immunoblots β-catenin signals were normalized to the loading markers and compared to controls, which were set to 100%. Means ± SEM (n = 3) are shown. Student's unpaired *t*-test compares Cd^2+ ^treated cells to respective controls. (B) Cd^2+^-induced TCF transcriptional activity depends on the confluence of kidney PTC. WKPT-0293 Cl.2 cells transfected with TOPflash, FOPflash or ΔN-β-catenin were treated with 25 μM Cd^2+ ^for 6 h. Data presented are means ± SEM of 4-5 experiments. Student's unpaired *t*-test compares Cd^2+ ^treated or ΔN-β-catenin-transfected cells to respective controls. (C) To determine association of TCF4 with β-catenin in ~50% and 100% confluent cells, nuclear fractions were immunoprecipitated with an antibody against β-catenin and immunoblotted for TCF4. (D) Expression of E-cadherin in whole cell lysates of ~50% and 100% confluent cells by immunoblotting.

To assess the activation of the Wnt transcriptional machinery upon Cd^2+ ^exposure, we performed luciferase activity measurements using the TOPflash reporter construct, which contains optimal TCF binding sites, in sub-confluent WKPT-0293 Cl.2 cells. Cd^2+ ^maximally increased TOPflash activity starting at 3-6 h but terminated at 12 h. After 6 h, the TCF reporter activity was 1.81 ± 0.21-fold (means ± SEM of 8 experiments; *P *= 0.009) (Fig. 2C). FOPflash activity, a negative control, was not affected by Cd^2+ ^(data not shown). For comparison, transfection of PTC with a ΔN β-catenin, which is resistant to proteasomal degradation [30] resulted in a 5.7 ± 1.5 fold (means ± SEM of 4 experiments; *P *= 0.02) increase of luciferase activity, compared to the control vector (Fig. 3B). The expression of Wnt signaling target genes involved in cell proliferation and survival, *c-Myc*, *cyclin D1 *and *Abcb1a*, was also increased by Cd^2+ ^exposure for up to 12 h (Fig. 2D). Experiments using EMSAs showed binding of TCF4 to the promoter sequence of both *ABCB1 *(Fig. 2E) and *cyclin D1 *(Additional file 3b) that was enhanced by Cd^2+ ^for up to 9 h, but peaked at 3 h. In order to assure that β-catenin was responsible for the transcription of target genes via the β-catenin/TCF4 transcription factor complex, we over-expressed wild-type hTCF4 and mutant ΔN-hTCF4. This mutant form of TCF4 lacks the NH_2_-terminal region required for interaction with β-catenin [25]. Cd^2+ ^(25 μM for 3-6 h) increased the expression of *c-Myc *in wild-type TCF4 over-expressing cells more profoundly than in vector-transfected or ΔN-hTCF4 mutant transfected cells (Fig. 2F). This confirms the role of β-catenin in mediating the up-regulation of Wnt target genes under Cd^2+ ^exposure of kidney PTC.

### Cd^2+^-induced Wnt signaling is triggered in non-confluent and proliferating but not in confluent and quiescent PTC

Interestingly, we found that the Wnt response activated by Cd^2+ ^is dependent on the confluence and proliferation status of the cells. With 100% confluent cells, we did not detect β- and α-catenin translocation to the cytosol and nuclei of Cd^2+^-exposed PTC, in contrast to ~50% confluent cells (Fig. 3A). This was associated with markedly reduced TOPflash activity. As shown in Fig. 3B, there was increased nuclear β-catenin bound to TCF4 in ~50% confluent cells but not in 100% confluent cells exposed to Cd^2+^. Moreover, ~50% confluent cells exhibited an ~2-fold increase in TOPflash transcriptional activity upon exposure to 25 μM Cd^2+ ^for 6 h (*P *= 0.007), whereas 100% confluent cells were not responsive (Fig. 3C). We also observed higher E-cadherin expression in 100% confluent than in ~50% confluent cells (Fig. 3D). Since β- and α-catenin are associated with E-cadherin at the AJ, it could account for the observed dependency of Cd^2+ ^to induce Wnt signaling on confluence.

### Role of E-cadherin expression levels in Cd^2+^-induced nuclear translocation of β-catenin and cell fate

**Figure 4:**
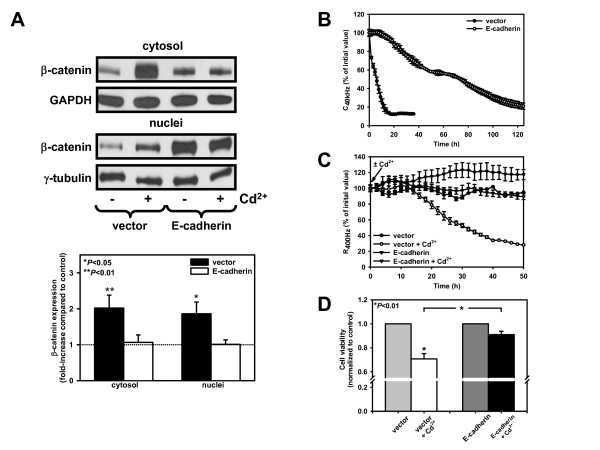
**E-cadherin overexpression in kidney PTC abolishes β-catenin redistribution, decreases proliferation and protects against Cd^2+^-induced epithelial barrier disruption and cytotoxicity**. (*A*)β-catenin immunoblots of WKPT-0293 Cl.2 cells transiently transfected with full length E-cadherin expression plasmid (E-cadherin) or empty vector (vector) and incubated for 6 h ± 25 μM Cd^2+^. β-catenin signals were normalized to loading markers and the ratio of β-catenin in Cd^2+ ^and respective control samples was determined. Means ± SEM (n = 7) are shown. Student's unpaired *t*-test compares Cd^2+ ^treated cells to respective controls. (B) Vector or E-cadherin PTC were reseeded in SCM in ECIS 8WE10 arrays. C_40 kHz _reflects cell attachment, spreading and proliferation (see Methods). Initial values of the cell-free electrode were set to 100% (vector, 63.5 ± 1.9 nF; E-cadherin, 65.2 ± 3.3 nF; means ± SEM of 3 experiments). Data show means ± SEM of 3 experiments. (C) At confluence PTC were exposed to SFM ± 20 μM Cd^2+^. Values of the confluent cell-covered electrode were set to 100% (vector, 3670 ± 83 ohm; E-cadherin, 2994 ± 300 ohm; means ± SEM of 3 different experiments). A drop in R_400 Hz _indicates disruption of epithelial barrier and cell detachment. (D) Vector or E-cadherin cells were exposed to 25 μM Cd^2+ ^for 6 h and cell viability was determined by MTT assay. Graph depicts means ± SEM of 9-10 experiments. Student's unpaired *t*-test compares Cd^2+ ^treated cells to respective controls as well as Cd^2+^-exposed vector to Cd^2+^-exposed E-cadherin cells.

To test the hypothesis that high levels of E-cadherin could prevent nuclear β-catenin translocation and Wnt signaling induced by Cd^2+^, we overexpressed full-length E-cadherin in WKPT-0293 Cl.2 cells. E-cadherin expression was increased by ~70% and was found to be uniformly distributed at the cell borders (Additional file 4a). When cells were subjected to 25 μM Cd^2+ ^for 6 h, the increase in cytoplasmic and nuclear β-catenin induced by Cd^2+ ^was abolished in E-cadherin overexpressing cells (Fig. 4A).

Because Cd^2+^-induced β-catenin translocation to the nucleus triggers β-catenin/TCF4-mediated Wnt signaling and increases expression of cell proliferation genes (see Fig. 2), we investigated the impact of E-cadherin on cell proliferation by ECIS. The ECIS technique measures the electrical properties of epithelial monolayers due to cell attachment/spreading/proliferation and barrier formation. C_40 kHz _reflects attachment, spreading and proliferation of cells whereas R_400 Hz _replicates epithelial barrier integrity and establishment of tight intercellular contacts (see Methods). C_40 kHz _for control cells decreased from values of the cell-free electrode to its baseline values (8-10 nF) after ~20 h (Fig. 4B), reflecting the establishment of an intact monolayer with coverage of the whole electrode surface. In contrast, E-cadherin over-expression in WKPT-0293 Cl.2 cells significantly retarded cell proliferation. Interestingly, however, once a stable monolayer was established, E-cadherin over-expressing cells were less sensitive to Cd^2+ ^(Fig. 4C). The decrease of R_400 Hz_, indicative of a disruption of the epithelial barrier, became apparent within hours after addition of 20 μM Cd^2+ ^in serum-free medium in vector-transfected cells, but proceeded far more slowly in E-cadherin over-expressing cells. Corresponding ΔC_40 kHz _increased by 1.76 ± 0.20 nF/h in vector-transfected cells as opposed to 0.91 ± 0.35 nF/h in E-cadherin-transfected PTC (means ± SEM of 3 experiments) reflecting increased detachment of cells from the electrode. This suggested that E-cadherin over-expression not only protects the epithelial monolayer from Cd^2+^-induced disruption of cellular junctions but also from detachment and death.

This was further tested by investigating the impact of E-cadherin overexpression on cell viability of WKPT-0293 Cl.2 cells exposed to Cd^2+^. Transfected cells were treated with 25 μM Cd^2+^, assessed using the MTT assay. Following treatment with Cd^2+^, cell viability decreased by ~30% in vector-transfected cells. Conversely, Cd^2+ ^could decrease cell viability by only ~9% in E-cadherin over-expressing cells (*P *< 0.01) (Fig. 4D). Taken together, these data indicate that E-cadherin overexpression decreases Wnt signaling-mediated cell proliferation and in confluent PTC monolayers is protective against Cd^2+^-induced disruption of the cellular junctions, detachment and cell death.

### Cd^2+^-induced ROS formation increases CHOP expression but does not antagonize Wnt signaling

**Figure 5:**
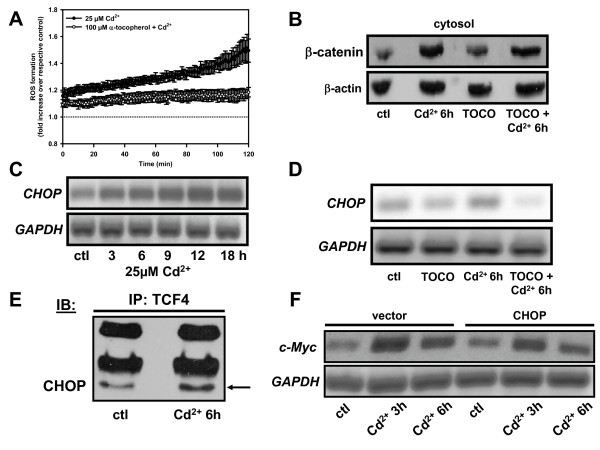
**Cd^2+^-induced reactive oxygen species (ROS) formation in kidney PTC induces up-regulation of CHOP, but has no effect on Wnt signaling**. (A) When used, the ROS scavenger α-tocopherol was pre-incubated for 1 h. Cells were loaded with 20 μM carboxy-H_2_DCFDA. Cell suspensions were incubated with Cd^2+ ^± α-tocopherol and fluorescent signals were measured immediately at 500/535 nm. Graph shows means ± SEM of 4-7 experiments. (B) β-catenin immunoblot of WKPT-0293 Cl.2 cells ± α-tocopherol (TOCO) (100 μM) ± 25 μM Cd^2+ ^for 6 h. (C) Expression of *CHOP *mRNA in WKPT-0293 Cl.2 cells without (ctl) or with 25 μM Cd^2+ ^by RT-PCR. (D) Prevention of Cd^2+^-mediated increase in *CHOP *mRNA expression by α-tocopherol. (E) Association of CHOP with TCF4 is increased in WKPT-0293 Cl.2 cells incubated with 25 μM Cd^2+ ^for 6 h when compared to controls (ctl). (F) Effect of CHOP overexpression on Cd^2+^-induced expression of *c-Myc *by RT-PCR. PTC transiently transfected with full length human CHOP (CHOP) or empty vector (vector) were exposed to 25 μM Cd^2+^.

ROS can be generated by Cd^2+ ^[31] and are known to disrupt TJs and AJs resulting in decreased transepithelial electrical resistance and redistribution of β-catenin [32]. Thus, we investigated whether the β-catenin accumulation in cytosol and nuclei is ROS-dependent. As shown in Fig. 5A, Cd^2+ ^(25 μM) caused an increase in ROS within minutes after Cd^2+ ^exposure which was determined by oxidation of the fluorescent probe carboxy-H_2_-DCFDA. ROS formation steadily increased over time reaching 30 ± 9% above controls at 2 h exposure (*P *< 0.01; n = 3), which was prevented by the antioxidant α-tocopherol (100 μM) (Fig. 5A). But Cd^2+ ^exposure showed similar effects on β-catenin distribution into the cytosol of PTC whether in the presence or absence of α-tocopherol (Fig. 5B), indicating that β-catenin release from the membrane and E-cadherin-bound pool to the intracellular space is ROS-independent.

CHOP, also known as GADD153, is a pro-apoptotic protein that is involved in the endoplasmic reticulum (ER) stress pathway. Increased expression of CHOP has been previously reported in the presence of Cd^2+ ^in kidney PTC as a function of ROS formation [33]. Moreover, CHOP has been previously shown to negatively regulate the Wnt signaling pathway through binding to TCF4 [34]. We also found that Cd^2+ ^treatment (25 μM) of WKPT-0293 Cl.2 cells increased the expression of CHOP in a time dependent manner (up to 18 h) (Fig. 5C), which was abrogated in the presence of α-tocopherol (Fig. 5D). CHOP was pulled down with TCF4 in immunoprecipitation experiments confirming CHOP-TCF4 interaction. This was further enhanced by Cd^2+ ^exposure (25 μM for 6 h) (Fig. 5E). Next, we investigated the expression of the Wnt target gene *c-Myc *in cells over-expressing CHOP. When treated with Cd^2+^, CHOP-overexpressing cells still showed an increment of *c-Myc *expression, indicating that CHOP does not prevent Cd^2+^-induced Wnt signaling (Fig. 5F). Cell viability studies with Cd^2+ ^for 6 h (Additional file 4b) or 12 h (data not shown) showed no effect of CHOP overexpression on Cd^2+^-induced cell death in PTC. Similarly, measurements of cell proliferation demonstrated that CHOP overexpression had no effect on proliferation in Cd^2+^-exposed cells (data not shown). Both findings are in accordance with the *c-Myc *expression data (Fig. 5F). From these data, we concluded that CHOP plays no significant role in preventing cell proliferation and survival of PT cells following Cd^2+ ^exposure though CHOP does bind to TCF4 (see Fig. 5E).

### Cd^2+ ^increases TCF4 expression which may override CHOP inhibition of Wnt signaling

**Figure 6:**
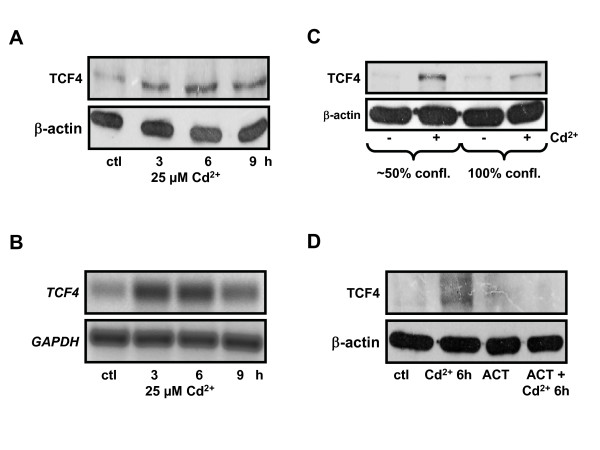
**Cd^2+ ^triggers TCF4 up-regulation in kidney PTC**. (A) TCF4 protein expression was increased by Cd^2+ ^in whole cell lysates from PTC. (B) RT-PCR of mRNA was performed with primers specific for rat *TCF4 *in WKPT-0293 Cl.2 cells without (ctl) or with Cd^2+^. Cd^2+ ^increased *TCF4 *gene expression. (C) TCF4 expression in whole cell lysates of ~50% confluent and 100% confluent cells ± Cd^2+ ^(25 μM for 6 h) by immunoblotting confirmed TCF4 up-regulation induced by Cd^2+ ^in subconfluent cells. (D) Actinomycin D abolished Cd^2+^-induced TCF4 up-regulation. PTC were treated with or without Cd^2+ ^(25 μM) ± actinomycin D (ACT) (10 μg/ml, 1 h pre-incubation), a transcriptional inhibitor. Expression of TCF4 protein was determined in whole cell lysates of WKPT-0293 Cl.2 cells by immunoblotting.

A possible reason for the discrepancy between our data and the literature may be the striking observation of increased total TCF4 elicited by Cd^2+ ^exposure from an early time point of 3 h up to 9 h Cd^2+ ^at the protein (Fig. 6A) and mRNA (Fig. 6B) levels. Moreover, TCF4 up-regulation by Cd^2+ ^in sub-confluent cells was strongly reduced in confluent monolayers (Fig. 6C). To strengthen our observation that TCF4 up-regulation by Cd^2+ ^is at the transcriptional level, we used the transcriptional inhibitor actinomycin D. In the presence of actinomycin D (10 μg/ml, pre-incubated for 1 h), Cd^2+ ^treatment failed to up-regulate TCF4 protein, when compared to cells treated with Cd^2+ ^alone (Fig. 6D). Hence increased TCF4 expression may override CHOP inhibition.

In contrast to the effect of Cd^2+ ^on TCF4, additional transcriptional components of Wnt/β-catenin signaling were not up-regulated by Cd^2+ ^exposure in sub-confluent PTC. *TCF3 *mRNA levels were similar in control and Cd^2+ ^treated cells for up to 18 h (Additional file 4c; *TCF3*). *LEF1*, a marker of mesenchymal cells [35], could not be detected, indicating the epithelial characteristic of the cell line and absence of any epithelial-to-mesenchymal transformation (EMT) phenomena upon Cd^2+ ^treatment (data not shown). Similarly, no change was detected in E-cadherin mRNA expression which is suppressed during EMT via the Wnt pathway (reviewed in [36]) (Additional file 4c; *E-cadherin*).

## Discussion

**Figure 7:**
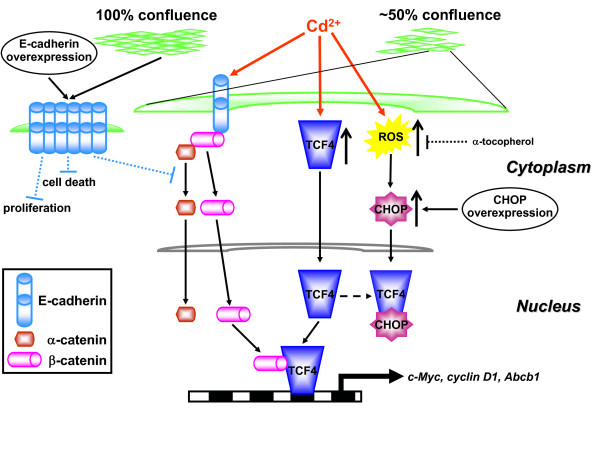
**Model for the effects of Cd^2+ ^on the adherens junction complex and Wnt signaling in WKPT-0293 Cl.2 kidney PTC**. For further details, see discussion.

To date, only one other transition metal ion, iron, has been reported to modulate the Wnt pathway [37]. However, this observation was made in cancer cell lines with non-functional APC or β-catenin mutations. In this study, we have described the activation of Wnt signaling by the carcinogen, Cd^2+ ^in PTC. Specifically, Cd^2+ ^induces redistribution, but not upregulation, of β-catenin from disrupted AJs to the nucleus where it forms a complex with TCF4 to up-regulate a number of proto-oncogenes, including *c-Myc*, *cyclin D1 *and *ABCB1*. A very intriguing observation is the dependence of this Cd^2+^-induced Wnt signaling on cell confluence, which could be explained by the augmented levels of E-cadherin in confluent cells that prevent β-catenin translocation. Both TCF4 and β-catenin orchestrate the transcription of Wnt target genes which promote proliferation and survival of affected cells. Thus we demonstrate induction of a novel pathway that could contribute to Cd^2+ ^carcinogenesis in pre-neoplastic renal cells (Fig. 7).

In the absence of a Wnt signal, β-catenin is mostly associated with the AJ complex of epithelial cells [38]. The turnover of β-catenin is tightly regulated by the Axin-APC-GSK3-β complex, which phosphorylates β-catenin and reinforces its degradation by the ubiquitin-proteasome pathway [39]. Mutations of components of the Wnt pathway increasing signaling lead to cancers of epithelial tissues (reviewed in [9]). For example, mutations in the *β-catenin*/*CTNNB1 *gene have been reported in aggressive fibromatosis (also called desmoid tumor) and parathyroid tumors [40,41] and mutations in the APC gene are often found in colon cancers. Both lead to the accumulation of β-catenin in the cytoplasmic pool, since the destruction complex fails to prime β-catenin for degradation [42]. To our knowledge, no such mutations are present in our model cell line that could account for increased β-catenin stabilization in Cd^2+^-treated WKPT-0293 Cl.2 cells.

Alpha-catenin is another important component of AJs and is believed to have tumor suppressor functions, possibly by negatively regulating β-catenin mediated Wnt signaling [29]. In this study, α-catenin was released into the cytosol by Cd^2+ ^exposure in kidney PTC, which is in line with other findings [43], and furthermore, we could demonstrate its translocation to the nuclei (Fig. 1C). However, reduced physical association between β-catenin and α-catenin was observed (Fig. 1D), suggesting that free rather than complexed β-catenin predominantly translocates to the nucleus. As β-catenin exists in the cells in at least five different distinct forms [44] it will be important to identify the form of β-catenin involved in the Cd^2+^-induced Wnt response.

Aberrant expression of TCF4 is associated with various forms of cancer, including renal cell carcinoma (RCC) [45,46]. It was intriguing to observe that Cd^2+ ^could induce transcription of TCF4 (Fig. 6) leading to increased accumulation of TCF4 in the nucleus. Little information is available about the transcriptional regulation of TCF4 apart from a report by Saegusa et al. [46] which showed in endometrial carcinoma cells that β-catenin can directly induce transcription from the TCF4 promoter in a positive feed-back loop, with the effect being enhanced by the p300 co-activator. Whether this mechanism is also operative in Cd^2+^-induced TCF4 up-regulation warrants future investigation.

Interestingly, sub-confluent cells were more responsive to Cd^2+ ^effects than confluent cells (Fig. 3). Confluent PTC revealed higher amounts of E-cadherin than sub-confluent cells, which can be explained by the fact that the former develop more cell-cell contacts and is in accordance with observations made in other cell systems [47]. E-cadherin repression or disruption is associated with cancer progression, invasiveness and is a key event in EMT [48]. On the other hand, E-cadherin expression is known to limit the degree and duration of Wnt/β-catenin signaling and EMT, possibly by increasing the turnover of cytosolic β-catenin due to the activity of an AJ-localized β-catenin phosphodestruction complex [47,49]. E-cadherin binding to β-catenin prevents nuclear localization of β-catenin and β-catenin/LEF-1-mediated transactivation [28,47]. In E-cadherin overexpressing cells, Cd^2+^-induced translocation of β-catenin was prevented (Fig. 4A) and cell proliferation of PTC was slowed down (Fig. 4B), which is in accordance with previous studies [50], and Cd^2+ ^cytotoxicity was reduced (Fig. 4C). Suppression of anoikis, a form of programmed cell death triggered by complete loss of anchorage, has been suggested to account for the resistance of E-cadherin overexpressing cells to detachment and death [51] and may contribute to our observations.

The E-cadherin/β-catenin interaction is largely dominated by phospho-regulation where phosphorylation of E-cadherin by kinases, such as the Src family of kinases, decreases β-catenin binding affinity (reviewed in [52]). Cd^2+ ^is known to induce a rapid activation of c-Src in mesangial cells [53], which is probably mediated by ROS formation [54]. Although Cd^2+ ^can generate ROS in PTC [31,33] (Fig. 5A), the antioxidant α-tocopherol failed to prevent the effects of Cd^2+ ^on β-catenin translocation (Fig. 5B) indicating that this phenomenon is rather caused by direct disruption of AJs by Cd^2+^, as first described by Prozialeck and coworkers (reviewed in [16]).

Cd^2+ ^has recently been shown to induce apoptosis of LLC-PK1 PTC through ROS formation and induction of ER stress [33], where both branches of the pro-apoptotic unfolded protein response, the IRE1-XBP1 and ATF6-CHOP pathways, were involved. The CCAAT/enhancer-binding protein-homologous protein (CHOP) (also called growth arrest and DNA damage inducible protein 153 (GADD153)) is a cell death marker that can act as Wnt repressor [34] and is expressed during ER stress of LLC-PK1 cells induced by Cd^2+ ^[55]. We could confirm CHOP induction by Cd^2+ ^in WKPT-0293 Cl.2 cells (Fig. 5C). Although we found increased CHOP binding to TCF4 upon Cd^2+ ^exposure of WKPT-0293 Cl.2 cells (Fig. 5E), CHOP over-expression failed to prevent Cd^2+^-induced Wnt signaling. This could possibly be due to the elevated levels of TCF4 (Fig. 6) that could surpass the inhibitory effects of CHOP.

From a clinical perspective, recent studies have shown that renal ischemia can also trigger β-catenin/Wnt signaling to promote survival of PTC, in part by inhibiting Bax in a phosphatidylinositol-3 kinase/Akt-dependent manner [56]. The carcinogenicity and toxicity of Cd^2+ ^has long been recognized [10] and could involve inactivation of the tumor suppressor p53 [57] or oxidative stress and inhibition of DNA repair (reviewed in [58]). Being a key signaling pathway for proliferation, the Wnt pathway could contribute to Cd^2+ ^nephrocarcinogenesis by creating growth advantage [14]. Cd^2+^-induced ROS formation also triggers pathways (e.g. FOXO, NF-κB, HIF-1α, CHOP), which may cross-talk with the Wnt/β-catenin pathway and are involved in life-and-death decisions or adaptation to stress-induced damage [59]. We previously showed that Cd^2+ ^triggers ABCB1 up-regulation in kidney PT cells which was partly mediated by NF-κB activation and resulted in enhanced protection against Cd^2+^-induced apoptosis [60]. Interestingly, the recent report by Solanas et al. [47] indicates that E-cadherin not only controls the transcriptional activity of β-catenin but also that of NF-κB.

Accordingly, increased expression of Wnt target genes could contribute to increased proliferation, survival as well as decreased cell death of PT cells exposed to Cd^2+ ^(but also other forms of stress-induced damage) and thereby promote Cd^2+ ^carcinogenesis, since evasion of apoptosis is a critical step for the survival of damaged and mutated pre-neoplastic cells [61]. Hence, Cd^2+^-induced Wnt/β-catenin-TCF4 signaling opens a new avenue in understanding the mechanisms of carcinogenesis following chronic Cd^2+ ^exposure.

## Conclusions

For the first time, we demonstrate that Cd^2+ ^activates Wnt signaling in renal PT cells and that Cd^2+^-induced transcription of target genes, including *c-Myc*, *cyclin D1 *and *Abcb1*, is mediated by Wnt signaling. Interestingly, these effects depend on the cell density and proliferation status. Induction of the Wnt pathway by Cd^2+ ^occurs by means of translocation of β-catenin to the nucleus and increased expression of the transcription factor TCF4, which can be prevented by E-cadherin overexpression. Cd^2+^-induced Wnt signaling, however, is independent of ROS formation and the pro-apoptotic ATF6/CHOP ER stress pathway triggered by Cd^2+^. We propose that Cd^2+ ^may facilitate carcinogenesis of PT cells by inducing the Wnt pathway to promote proliferation and survival of pre-neoplastic cells.

## List of Abbreviations

ABCB1: ATP-binding cassette sub-family B member 1; AJ: adherens junctions; APC: adenomatous polyposis coli; ATF6: Activating transcription factor 6; Cd: cadmium; CHOP: C/EBP homologous protein; CK1-α: casein kinase 1-α; ECIS: electric cell-substrate impedance sensing; EMSA: electromobility shift assay; EMT: epithelial to mesenchymal transition; ER: endoplasmic reticulum; FAP: familial adenomatous polyposis; GADD153: growth arrest and DNA damage-inducible gene 153; GSK3-β: glycogen synthase kinase 3-β; MTT: 3-(4,5-dimethylthiazol-2-yl)-2,5-diphenyltetrazolium bromide; NF-κB: nuclear factor-kappa B; PTC: proximal tubule cells; RCC: renal cell carcinoma; ROS: reactive oxygen species; SCM: serum-containing medium; SFM: serum-free medium; TCF/LEF: T-cell factor/lymphoid enhancer factor; TJ: tight-junctions; VHL: von Hippel-Lindau.

## Competing interests

The authors declare that they have no competing interests.

## Authors' contributions

PKC carried out cell culture work, biochemical, molecular biological and immunofluorescence studies, performed statistical analyses and drafted parts of the manuscript. WKL carried out cell culture, transfection experiments, cell death assays, measurements of reactive oxygen species, statistical analyses and drafted parts of the manuscript. MM performed cell culture, transfections, participated in the biochemical and molecular biological experiments and statistical analyses. NAW performed cell culture, transfections, electrophysiological studies and statistical analyses and helped to draft parts of the manuscript. FT conceived the study, participated in its design and coordination, performed statistical analyses and helped to draft the manuscript. All authors read and approved the final manuscript.

## Supplementary Material

Additional file 1 **Primers for RT-PCR**. Primer sequences and GenBank accession numbers of the gene products tested are listed. Click here for file

Additional file 2 **Cd^2+ ^increases β-catenin distribution from the periphery to cytosol and nuclei of kidney PTC without affecting *β-catenin *gene expression**. (a) Expression of *β-catenin *and house-keeping gene *GAPDH *mRNA in WKPT-0293 Cl.2 cells without (ctl) or with Cd^2+ ^by RT-PCR. (b) β-catenin immunoblots showed no effect of the translational inhibitor cycloheximide (CHX) (20 μg/ml; 1 h preincubation) or (c) of the proteasomal inhibitor lactacystin (LACT) (1 μM; 1 h preincubation) on β-catenin redistribution in WKPT-0293 Cl.2 cells induced by Cd^2+^. Click here for file

Additional file 3 **Cd^2+ ^enhances nuclear TCF4/β-catenin binding and activity in kidney PTC**. (a) Increased binding of β-catenin to immunoprecipitated TCF4 in Cd^2+ ^treated WKPT-0293 Cl.2 cells. (b) EMSA analysis of TCF4 binding to *cyclin D1 *promoter region. Nuclear extracts of WKPT-0293 Cl.2 cells were incubated with [γ-^32^P]-end-labeled oligonucleotides containing the wild-type TCF4 binding sequence of the human *cyclin D1 *promoter region. Apart from controls (ctl) and Cd^2+ ^treated cells (25 μM for 3-9 h), lysate free sample (blank), and extract from cells exposed to Cd^2+ ^for 3 h incubated with a 200-fold excess of competing unlabeled oligonucleotides (comp.) were loaded. Binding of *cyclin D1 *promoter oligonucleotides to TCF4 was increased upon Cd^2+ ^exposure. Click here for file

Additional file 4 **E-cadherin expression on control and E-cadherin overexpressing PTC, effect of CHOP overexpression on Cd^2+ ^toxicity and effect of Cd^2+ ^on mRNA expression of EMT markers in PTC**. (a) Protein expression and immunofluorescence staining pattern of E-cadherin in WKPT-0293 Cl.2 cells transiently transfected with E-cadherin (E-cadherin) or empty vector pL31NU (vector) for 36 h. Note the increased peripheral β-catenin labeling in E-cadherin-overexpressing cells. Bars = 20 μm. (b) CHOP overexpression does not affect Cd^2+ ^toxicity. WKPT-0293 Cl.2 cells were transfected with empty vector or CHOP overexpressing plasmid followed by incubation with 25 μM Cd^2+ ^for 6 h. Cell viability was determined by the MTT assay and data were normalized to respective controls. MTT absorbance was similar in empty vector (0.34 ± 0.04) and CHOP (0.35 ± 0.03) transfected cells. Graphs depict means ± SEM of 7 experiments. Student's unpaired *t*-test compares Cd^2+ ^treated cells to respective controls as well as Cd^2+^-exposed vector-transfected to Cd^2+^-exposed and CHOP-transfected cells. n.s. = not significant. (c) RT-PCR with primers specific for rat *TCF3*, *E-cadherin *or *GAPDH *in WKPT-0293 Cl.2 cells ± Cd^2+^. Cd^2+ ^had no effect on *TCF3, E-cadherin *or *GAPDH *mRNA. Click here for file
