# Supplementary material for: Cadmium induces Wnt signaling to upregulate proliferation and survival genes in sub-confluent kidney proximal tubule cells
Source: Mol Cancer. 2010 May 8;9:102. doi: 10.1186/1476-4598-9-102 (PMC2873433; doi:10.1186/1476-4598-9-102)
Supplement: Additional file 1 — Primers for RT-PCR. Primer sequences and GenBank accession numbers of the gene products tested are listed. [file 1476-4598-9-102-S1.PDF]

Gene	Forward primer	Reverse Primer	GenBank Accession Number
LEF-1	5’-TAACAAGGGCCCCTCCTACT-3’	5’-CCTGGAGAAAAGTGCTCGTC-3’	NM_130429.1
TCF-3	5’-CAGTCTCAGCAGCAAATCCA-3’	5’-GGCTTGCTCGGAGTGAGTAG-3’	NM_001107865.1
TCF-4	5’-CTAGGCGCTAACGACGAACT-3’	5’-CCGGGATTTATCTCGGAAAC-3’	XM_001072361.1
E-cadherin	5’-GTGCCTGAGGACTTTGGTGT-3’	5’-ACGTGCTGTTCTTCACATGC-3’	NM_031334.1
β-catenin	5’-GCCAGTGGATTCCGTACTGT-3’	5’-GAGCTTGCTTTCCTGATTGC-3’	AF397179.1
Cyclin D1	5’-TCAGATGTCCACATCTCGGACGTCG-3’	5’-ATGGAACACCAGCTCCTGTGCTGCG-3’	NM171992
ABCB1	5’-GATGGAATTGATAATGTGGACA-3’	5’-AAGGATCAGGAACAATAAA-3’	NM_133401.1
CHOP	5’-CCTGAAAGCAGAAACCGGTC-3’	5’-CCTGAAAGCAGAAACCGGTC-3’	NM_024134
