# Supplementary figures and images for: Cadmium induces Wnt signaling to upregulate proliferation and survival genes in sub-confluent kidney proximal tubule cells
Source: Mol Cancer. 2010 May 8;9:102. doi: 10.1186/1476-4598-9-102 (PMC2873433; doi:10.1186/1476-4598-9-102)

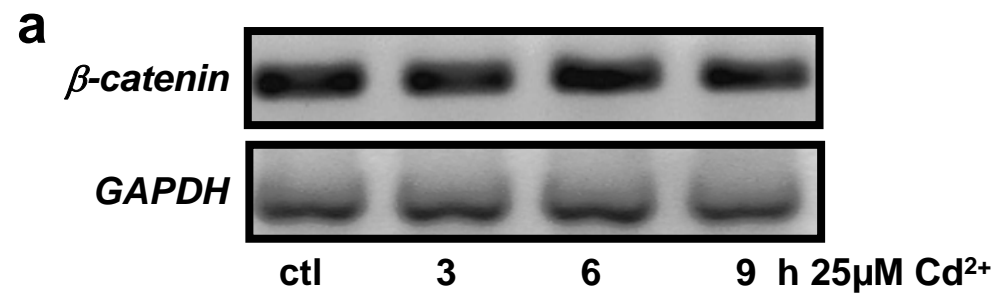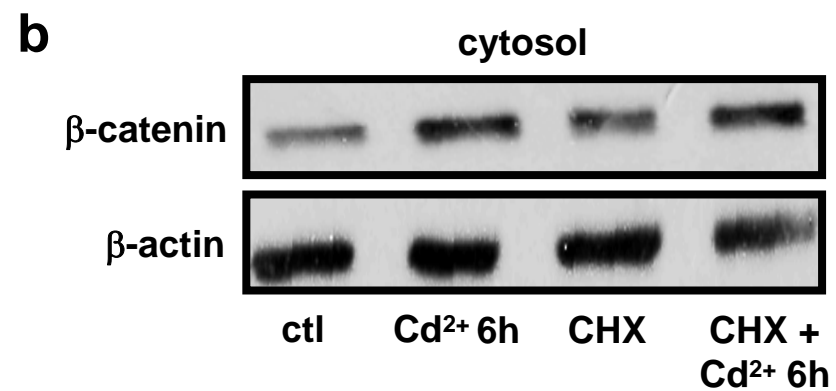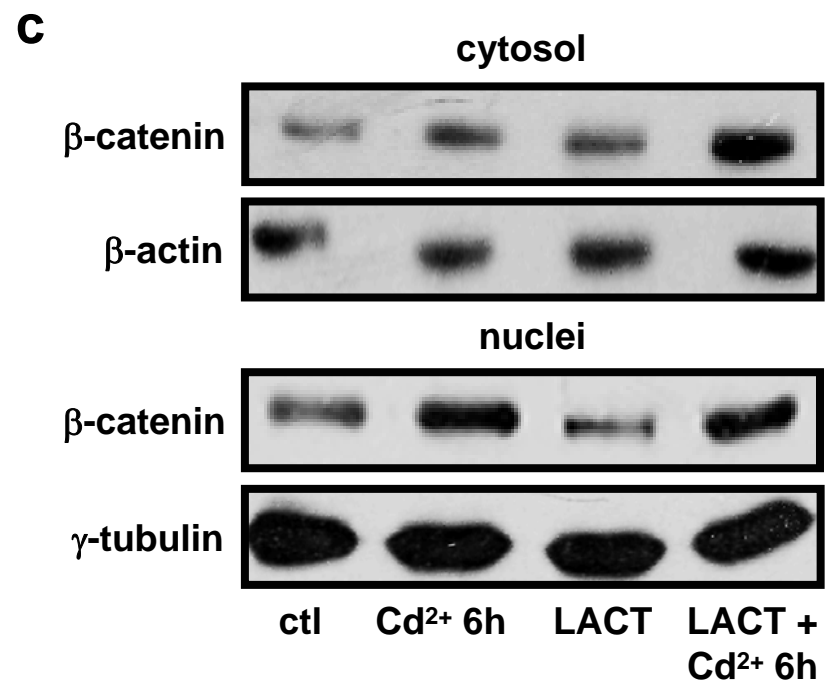

Supplement: Additional file 2 — Cd2+ increases β-catenin distribution from the periphery to cytosol and nuclei of kidney PTC without affecting β-catenin gene expression. (a) Expression of β-catenin and house-keeping gene GAPDH mRNA in WKPT-0293 Cl.2 cells without (ctl) or with Cd2+ by RT-PCR. (b) β-catenin immunoblots showed no effect of the translational inhibitor cycloheximide (CHX) (20 μg/ml; 1 h preincubation) or (c) of the proteasomal inhibitor lactacystin (LACT) (1 μM; 1 h preincubation) on β-catenin redistribution in WKPT-0293 Cl.2 cells induced by Cd2+. [file 1476-4598-9-102-S2.PDF]

**a**

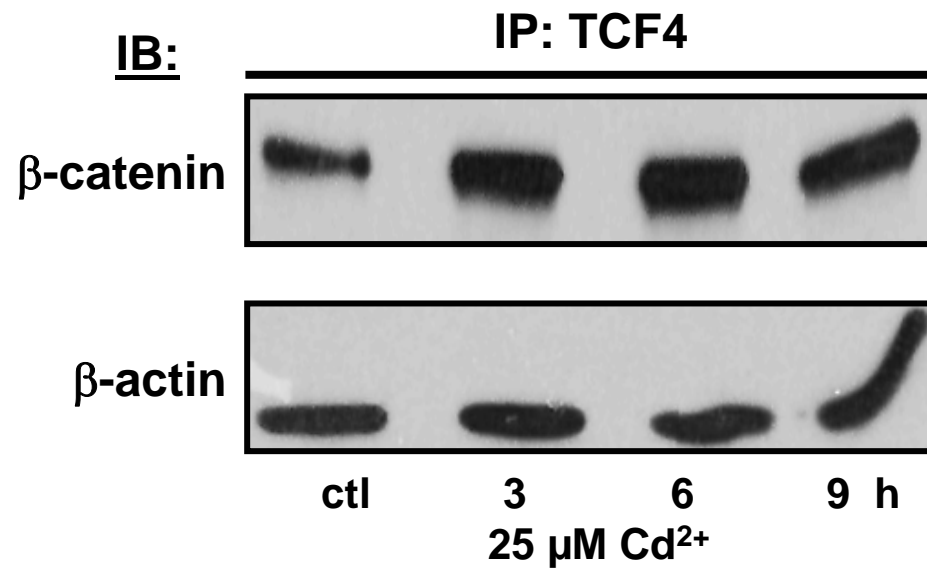

**b**

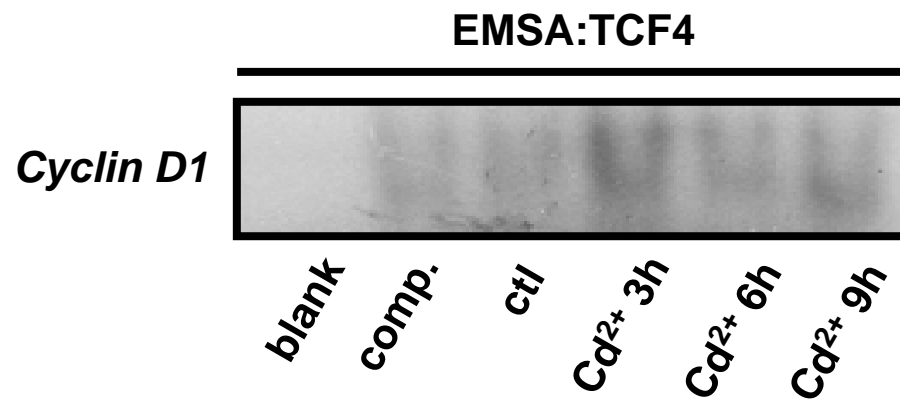

Supplement: Additional file 3 — Cd2+ enhances nuclear TCF4/β-catenin binding and activity in kidney PTC. (a) Increased binding of β-catenin to immunoprecipitated TCF4 in Cd2+ treated WKPT-0293 Cl.2 cells. (b) EMSA analysis of TCF4 binding to cyclin D1 promoter region. Nuclear extracts of WKPT-0293 Cl.2 cells were incubated with [γ-32P]-end-labeled oligonucleotides containing the wild-type TCF4 binding sequence of the human cyclin D1 promoter region. Apart from controls (ctl) and Cd2+ treated cells (25 μM for 3-9 h), lysate free sample (blank), and extract from cells exposed to Cd2+ for 3 h incubated with a 200-fold excess of competing unlabeled oligonucleotides (comp.) were loaded. Binding of cyclin D1 promoter oligonucleotides to TCF4 was increased upon Cd2+ exposure. [file 1476-4598-9-102-S3.PDF]

**a**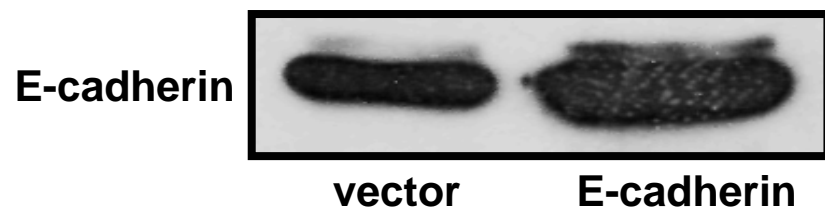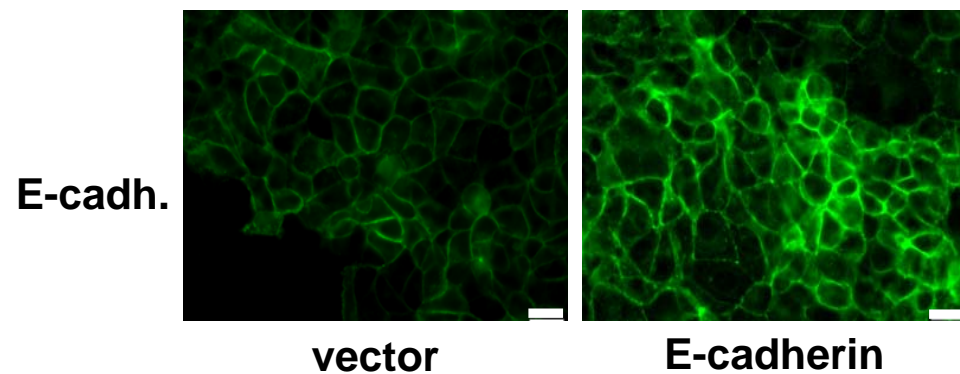**b**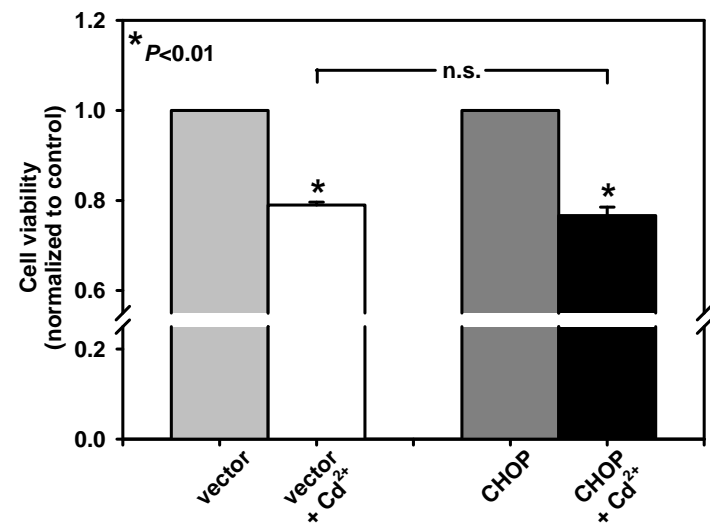**c**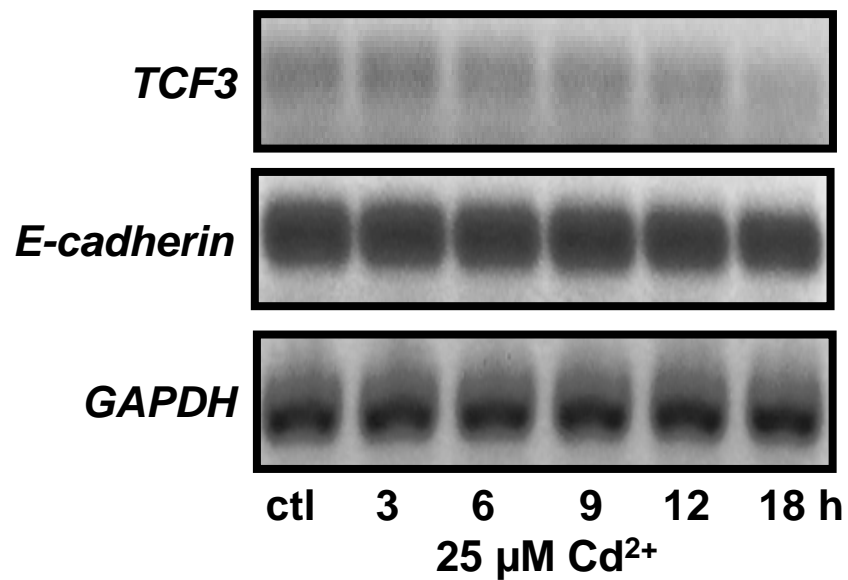

Supplement: Additional file 4 — E-cadherin expression on control and E-cadherin overexpressing PTC, effect of CHOP overexpression on Cd2+ toxicity and effect of Cd2+ on mRNA expression of EMT markers in PTC. (a) Protein expression and immunofluorescence staining pattern of E-cadherin in WKPT-0293 Cl.2 cells transiently transfected with E-cadherin (E-cadherin) or empty vector pL31NU (vector) for 36 h. Note the increased peripheral β-catenin labeling in E-cadherin-overexpressing cells. Bars = 20 μm. (b) CHOP overexpression does not affect Cd2+ toxicity. WKPT-0293 Cl.2 cells were transfected with empty vector or CHOP overexpressing plasmid followed by incubation with 25 μM Cd2+ for 6 h. Cell viability was determined by the MTT assay and data were normalized to respective controls. MTT absorbance was similar in empty vector (0.34 ± 0.04) and CHOP (0.35 ± 0.03) transfected cells. Graphs depict means ± SEM of 7 experiments. Student's unpaired t-test compares Cd2+ treated cells to respective controls as well as Cd2+-exposed vector-transfected to Cd2+-exposed and CHOP-transfected cells. n.s. = not significant. (c) RT-PCR with primers specific for rat TCF3, E-cadherin or GAPDH in WKPT-0293 Cl.2 cells ± Cd2+. Cd2+ had no effect on TCF3, E-cadherin or GAPDH mRNA. [file 1476-4598-9-102-S4.PDF]
